# Case Report: Reappearance of spermatozoa following multicomponent lifestyle modification in men with previously diagnosed azoospermia: a case series

**DOI:** 10.3389/fruro.2026.1903697

**Published:** 2026-09-28

**Authors:** Michael Enwere, Mohamed Toufic El-Hussein, Emmanuel Davies, Laurens Holmes

**Affiliations:** 1 PrimeLife360 Wellness, London, United Kingdom; 2 AgeCare Research Chair in Older Adults Health, School of Nursing and Midwifery, Faculty of Health, Community and Education, Mount Royal University, Calgary, AB, Canada; 3 Department of Cardiac Sciences, Cumming School of Medicine, University of Calgary, Calgary, AB, Canada; 4 Public Health Institute, FAMU, Tallahassee, FL, United States

**Keywords:** azoospermia, case series, male infertility, multicomponent lifestyle intervention, severe oligozoospermia, spermatogenesis, weight loss, whole-food plant-based (WFPB)

## Abstract

Azoospermia, defined as the absence of spermatozoa in the ejaculate, is a severe form of male infertility, and the potential effects of intensive lifestyle modification on sperm production remain uncertain. We describe three adult men whose available pre-intervention records reported azoospermia and who undertook a multicomponent lifestyle programme centred on a predominantly raw, whole-food, plant-based diet, prescribed hydration, progressive physical activity, sleep and stress-management guidance, reduced alcohol consumption, and avoidance of ultra-processed and animal-derived foods. Clinical records, serial semen analyses, and available hormonal assessments were retrospectively reviewed. Approximately 10 weeks after programme initiation, spermatozoa were detected in all three participants, with concentrations of 1.6, 2.0, and 2.2 × 10^6^/mL, respectively, at the first sperm-positive assessments; these values remained within a severe oligozoospermic range, and motility and subsequent trajectories varied. Two participants had previously received fertility-directed hormonal therapies, and one had undergone unsuccessful testicular sperm extraction. Participants also reported improvements in body weight, sleep, physical-activity tolerance, libido, perceived energy, and general well-being, with no major adverse events documented. Complete etiologic assessment, genetic testing, objective adherence measures, and uniform documentation of semen-analysis methods were not available for all participants. This uncontrolled case series documents a clinically notable temporal association between initiation of an intensive lifestyle programme and subsequent detection of spermatozoa in men with previously reported azoospermia. Causality cannot be established, and the individual contributions of diet and other programme components remain uncertain. Prospective controlled studies using standardized andrologic assessment, comprehensive etiologic investigation, objective adherence measures, and prespecified reproductive outcomes are warranted.

## Introduction

Approximately one in six people of reproductive age worldwide experience infertility during their lifetime (1). Azoospermia, defined as the absence of spermatozoa in the ejaculate, is one of the most severe forms of male infertility, affecting approximately 1% of men and 10–15% of men with infertility (2, 3). It is broadly classified as obstructive azoospermia (OA), in which spermatogenesis is preserved but sperm transport is impaired, or non-obstructive azoospermia (NOA), in which spermatogenesis itself is impaired (4, 5). This distinction has direct implications for prognosis, diagnostic evaluation, and treatment.

The causes of impaired spermatogenesis are heterogeneous. Genetic abnormalities, endocrine dysfunction, oxidative stress, mitochondrial dysfunction, inflammation, and metabolic dysregulation have all been implicated, although many cases remain idiopathic (3–12). Lifestyle and environmental factors may also affect male reproductive health. Poor diet quality, obesity, physical inactivity, smoking, alcohol exposure, and environmental toxicants have been associated with adverse reproductive and semen outcomes through overlapping metabolic, endocrine, inflammatory, oxidative, and gonadotoxic pathways (13–16). These associations do not show that lifestyle modification can restore spermatogenesis in azoospermia, but they support investigation of modifiable factors alongside standard andrologic care.

Management of azoospermia is determined by aetiology and may include correction of reversible causes, fertility-directed hormonal therapy in selected patients, surgical sperm retrieval, and assisted reproductive technologies. The role of structured lifestyle intervention as an adjunct remains poorly defined. Whole-food, plant-rich dietary patterns have been studied for their effects on cardiometabolic, inflammatory, endothelial, and gut-microbial pathways (17–20), but direct clinical evidence that such approaches, alone or within a broader lifestyle programme, alter sperm production in men with azoospermia remains limited.

The objective of this case series was to describe the clinical chronology, intervention exposure, and available semen findings. These observations establish temporal sequence only; the series was not designed to determine causality or isolate the effect of any individual programme component.

## Methods

### Study design and participants

This retrospective case series describes three adult men with pre-intervention records reporting azoospermia who undertook a structured lifestyle-medicine programme in clinical practice. Eligible participants were adults (≥18 years) with available documentation reporting no spermatozoa in the ejaculate and no recent surgery for a known obstructive cause. Individuals with active malignancy, acute systemic illness, or a documented irreversible genetic cause of infertility, where known, were excluded. Written informed consent was obtained for publication of de-identified clinical information.

### Lifestyle intervention

Participants undertook a structured programme centred on a predominantly raw, whole-food, plant-based dietary pattern, prescribed hydration, progressive physical activity, sleep and stress-management guidance, and avoidance of alcohol, animal-derived foods, refined sugars and ultra-processed foods. The programme was prescribed and supervised by the treating physicians during the intensive phase and emphasised leafy green and cruciferous vegetables and plant sources of omega-3 fatty acids. During the intensive phase, permitted raw vegetables could be consumed ad libitum, but the protocol specified minimum daily targets of 454 g of leafy green and cruciferous vegetables; either ½ cup of ground flaxseed or chia seeds, or 3 tablespoons of cold-pressed flaxseed oil; and approximately 3.0–3.8 L of water, adjusted for body weight and clinical status. Fruit was limited to approximately 25% of total food volume, estimated visually. The programme did not prescribe an individualised energy target or fixed macronutrient distribution; permitted foods were otherwise consumed ad libitum. Micronutrient supplementation was guided by available laboratory findings. Serum 25-hydroxyvitamin D was interpreted using the reporting laboratory’s reference interval of 31–100 ng/mL; below-range values prompted individualised vitamin D3–K2 supplementation. Serum vitamin B12 was not measured, but B12 supplementation was recommended because animal-derived foods were excluded. Participant-specific 25-hydroxyvitamin D values are presented in **Tables 1**–**3**.

**Table 1:** Longitudinal semen, endocrine, and microbiological findings for Case 1.

Laboratory parameter	05 Apr 2024 (Azoospermia)	25 Apr 2024 (Hormonal Evaluation)	01 July 2025 (Severe Oligozoospermia)	03 March 2026(Severe Oligozoospermia)	Reference Range / Interpretation
**Clinical Interpretation**	**Azoospermia**	**Hormonal Evaluation**	**Severe Oligozoospermia**	**Severe Oligozoospermia**	
Clinical trajectory: Azoospermia documented in April 2024 → hormonal assessment in April 2024 → spermatozoa detected as severe oligozoospermia in July 2025 during follow-up after initiation of the multicomponent lifestyle intervention → persistent severe oligozoospermia in March 2026.					
Semen parameters					
Sperm concentration	**Not detected**	—	**1.6 ×10^6^/mL**	**1.4 × 10^6^/mL**	**≥16 × 10^6^/mL**
Active (Progressive) Motility	**NA**	—	**5%**	**20%**	**≥30%**
Sluggish (Non-Progressive) Motility	**NA**	—	5%	**60%**	
Non-Motile Sperm	**NA**	—	**90%**	**20%**	
Normal Morphology	**NA**	—	**10%**	**70%**	
Double Head	**Not detected**		**10%**	**10%**	
Pin Head	**Not detected**		**20%**	**10%**	
Tail	**Not detected**		**60%**	**10%**	
Semen Volume (mL)	1.5 mL	—	5.0 mL	4.8 mL	
Semen pH	6.5	—	8.0	8.0	≥7.2
Liquefaction	50		50	35	30–60 min
Semen Fructose Qualitative	*Not available*		Positive	Positive	Positive
Hormonal profile					
FSH (mIU/mL)	—	8.05	4.8	—	
LH (mIU/mL)	—	—	4.2	—	
Total Testosterone (ng/mL)	—	**1.22**	**11.9**	—	
Prolactin (ng/mL)	—	**24.5**	**8.8**	—	
Microbiological assessment					
Culture/Microbiology	**No bacterial growth**	—	—	**No bacterial growth**	
25-hydroxyvitamin D	7 ng/mL				**31–100** ng/mL
Weight (Kg)		100.6 kg	88.0 kg		
BMI		34.8 kg/m²	30.5 kg/m²	Not available	

The hormonal values shown for 25 April 2024 and 1 July 2025 were transcribed exactly from the corresponding original external laboratory report. No values were approximated or imputed.

FSH, follicle-stimulating hormone; LH, luteinizing hormone. Reference ranges: FSH 1.5–12.4 mIU/mL; LH 1.7–8.6 mIU/mL; Total Testosterone 2.49–11.1 ng/mL (adult male); Prolactin 2.0–18.0 ng/mL.

Severe oligozoospermia is used descriptively for sperm concentration <5 × 10^6^/mL and is not a WHO 2021 diagnostic category. Azoospermia defined as absence of spermatozoa in the ejaculate after centrifugation. Boldface indicates key laboratory values or categorical findings highlighted for clinical interpretation; boldface does not indicate statistical significance.

Colour key: Red, no spermatozoa detected or value below the stated reference limit; amber, detectable value below the stated reference limit; green, value meeting the stated reference limit; blue, endocrine assessment; grey, parameter not assessed or not applicable. The table remains interpretable without reliance on colour.

Semen reference values identified as WHO-derived represent lower reference values reported in the sixth edition of the *WHO laboratory manual for the examination and processing of human semen* (2021) (23). These values describe the lower fifth centiles of semen distributions in a reference population and should not be interpreted as absolute diagnostic cutoffs or as thresholds separating fertile from infertile men. Reference intervals not identified as WHO-derived were reproduced from the corresponding external laboratory reports and may reflect laboratory-specific methods, reporting conventions and reference populations. NA indicates that motility or morphology assessment was not applicable because no spermatozoa were detected; an em dash (—) indicates that the parameter was not assessed. Missing measurements were not estimated or substituted.

FSH, follicle-stimulating hormone; LH, luteinizing hormone; NA, not applicable; WHO, World Health Organization. The table summarizes available laboratory findings before and during follow-up after initiation of the multicomponent lifestyle intervention. The temporal sequence does not establish that the intervention caused the subsequent semen findings.

**Table 2:** Longitudinal semen, endocrine, and microbiological findings for Case 2.

Laboratory parameter	16 Apr 2023 (Hormonal evaluation)	23 Jun 2023 (Azoospermia)	22 Mar 2026 (Severe oligozoospermia)	01 Apr 2026 (Microbiological assessment)	Reference range/Interpretation
**Clinical Interpretation**	**Hormonal Evaluation**	**Azoospermia**	**Severe Oligozoospermia**	**Positive seminal culture/Microbiology**	
Clinical trajectory: Baseline hormonal evaluation (Apr 2023) → azoospermia reported (Jun 2023) → spermatozoa detected at a concentration within the severe oligozoospermic range (Mar 2026) → Staphylococcus aureus isolated on seminal culture (Apr 2026) during follow-up after programme initiation.					
Semen parameters					
Sperm Concentration	—	**0.00 × 10^6^/mL**	**2.0 × 10^6^/mL**	—	*≥16* **× 10^6^/mL**
Progressive Motility	—	**NA**	**15%**	—	*≥30%*
Non-Progressive Motility	—	**NA**	**15%**	—	*—*
Non-Motile Sperm	—	**NA**	**70%**	—	*—*
Normal Morphology	—	**NA**	**20%**	—	*≥4%*
Abnormal Morphology	—	**NA**	**80%**	—	*—*
Semen Volume (mL)	—	1.5 mL	2.0 mL	—	*≥1.4 mL*
Semen pH	—	6.5	6.5	—	*≥7.2*
Leucocytes/Pus Cells	—	<1/hpf	**4–6/hpf**	**3–6/hpf**	*0–1/hpf*
Epithelial Cells	—	Absent	**++**	**+**	
Bacterial Presence (Microscopy)	—	Absent	**Bacterial (++)**	**Present**	*Absent*
Appearance/Color	—	Grey White	Creamy white	—	*Grey white/Creamy white*
Liquefaction	—	Complete	—	—	*Complete (<60 min)*
Viscosity	—	Non-viscous	Normal	—	*Non-viscous*
Hormonal profile					
Total Testosterone (ng/mL)	**2.18**	—	—	—	*2.2 – 10.5*
FSH (IU/L)	**6.15**	—	—	—	*1.3 – 19.3*
LH (mIU/mL)	**3.69**	—	—	—	*1.24 – 8.62*
Prolactin (mIU/L)	**102.3**	—	—	—	*53 – 360*
Microbiological assessment					
Culture Result	—	No bacterial growth	—	***Heavy growth Staphylococcus aureus***	*No growth*
Other Organisms	—	None	—	*None reported*	*None*
Antibiotic sensitivity (01 Apr 2026)					
**Sensitive**	Levofloxacin (+++), Augmentin (+++), Gentamycin (+)				—
**Resistant**	Sparfloxacin, Streptomycin, Pefloxacin, Rocephin, Zinacef, Ciprofloxacin, Azithromycin, Erythromycin, Co-trimoxazole				—
25-hydroxyvitamin D	16 ng/mL				31–100 ng/mL
Weight (Kg)	89.7 kg 81.7 kg				
BMI	29.3 kg/m² 26.7 kg/m²				

*No motile or non-motile sperm identified because spermatozoa were absent.

FSH, Follicle-stimulating hormone; LH, Luteinizing hormone; SFA, Seminal Fluid Analysis; hpf, high power field.

Severe oligozoospermia is used descriptively for sperm concentration <5 × 10^6^/mL and is not a WHO 2021 diagnostic category. Boldface indicates key laboratory values or categorical findings highlighted for clinical interpretation; boldface does not indicate statistical significance.

Colour key: Red, no spermatozoa detected or value below the stated reference limit; amber, detectable value below the stated reference limit; green, value meeting the stated reference limit; blue, endocrine assessment; grey, parameter not assessed or not applicable. The table remains interpretable without reliance on colour.

Semen reference values identified as WHO-derived represent lower reference values reported in the sixth edition of the WHO laboratory manual for the examination and processing of human semen (2021) (23). These values describe the lower fifth centiles of semen distributions in a reference population and should not be interpreted as absolute diagnostic cutoffs or as thresholds separating fertile from infertile men. Reference intervals not identified as WHO-derived were reproduced from the corresponding external laboratory reports and may reflect laboratory-specific methods, reporting conventions and reference populations. NA indicates that motility or morphology assessment was not applicable because no spermatozoa were detected; an em dash (—) indicates that the parameter was not assessed. Missing measurements were not estimated or substituted.

FSH, follicle-stimulating hormone; hpf, high-power field; LH, luteinizing hormone; NA, not applicable; SFA, seminal fluid analysis; WHO, World Health Organization. The initial semen assessment documented azoospermia. During follow-up after initiation of the multicomponent lifestyle intervention, a subsequent semen analysis detected spermatozoa within the severe oligozoospermic range. Later microbiological assessment demonstrated inflammatory findings and heavy growth of *Staphylococcus aureus*. The temporal sequence does not establish that the intervention caused the subsequent semen findings.

**Table 3:** Longitudinal semen, endocrine, and microbiological findings for Case 3.

Laboratory parameter	17 Feb 2023 (Severe oligozoospermia)	06 Mar 2023 (Azoospermia)	25 May 2026 (Severe oligozoospermia)	Microbiology (No growth)	Reference range/ Interpretation
**Clinical Interpretation**	**Severe Oligozoospermia**	**Azoospermia**	**Severe Oligozoospermia**	**No growth**	
***Clinical trajectory:*** *Severe oligozoospermia documented in February 2023 → azoospermia documented in March 2023 → spermatozoa detected as severe oligozoospermia in May 2026 during follow-up after initiation of the multicomponent lifestyle intervention.*					
Semen parameters					
Sperm Concentration	0.1 × 10^6^/mL	Not detected	2.2 × 10^6^/mL	—	≥16 × 10^6^/mL
Progressive Motility	6%	**NA**	35%	—	≥30%
Non-Progressive Motility	18%	**NA**	15%	—	—
Non-Motile Sperm	76%	**NA**	50%	—	—
Normal Morphology	**NA**	**NA**	20%	—	≥4%
Semen Volume (mL)	3.0 mL	5.2 mL	5.5 mL	—	≥1.4 mL
Semen pH	8.0	8.0	7.5	—	≥7.2
Hormonal profile					
Total Testosterone (ng/mL)	—	6.4 ng/mL	7.2 ng/mL	—	3.0–10.0 ng/mL
Prolactin (ng/mL)	—	1.3 ng/mL	6.5 ng/mL	—	3.0–19 ng/mL
Micorbiological assessment					
Culture Result	No pathogenic growth	No significant bacterial growth after 48 hrs	No bacterial growth identified	No bacterial growth identified	No growth
Other Organisms	None identified	None identified	None identified	None identified	None
25-hydroxyvitamin D		16 ng/mL			31–100 ng/mL
Weight (Kg)		86.8 kg	77.8 kg		
BMI		29.0 kg/m²	26.0 kg/m²	—	

*No spermatozoa were identified during the azoospermic assessment despite repeat evaluation.

Abbreviations: WHO, World Health Organization; hpf, high-power field. Severe oligozoospermia is used descriptively for sperm concentration <5 × 10^6^/mL and is not a WHO 2021 diagnostic category. Boldface indicates key laboratory values or categorical findings highlighted for clinical interpretation; boldface does not indicate statistical significance.

Colour key: Red, no spermatozoa detected or value below the stated reference limit; amber, detectable value below the stated reference limit; green, value meeting the stated reference limit; blue, endocrine assessment; grey, parameter not assessed or not applicable. The table remains interpretable without reliance on colour.

Semen reference values identified as WHO-derived represent lower reference values reported in the sixth edition of the World Health Organization Laboratory Manual for the Examination and Processing of Human Semen (2021) (23). These values represent the lower fifth centiles of semen-parameter distributions in the reference population and should not be interpreted as absolute diagnostic cutoffs or thresholds separating fertile from infertile men. Reference intervals not identified as WHO-derived were reproduced from the corresponding external laboratory reports and may reflect laboratory-specific methods, reporting conventions, and reference populations. NA indicates that motility or morphology assessment was not applicable because no spermatozoa were detected; an em dash (—) indicates that the parameter was not assessed. Missing measurements were not estimated or substituted.

NA, not applicable; WHO, World Health Organization. The initial assessment documented severe oligozoospermia, followed by an azoospermic assessment in March 2023. During follow-up after initiation of the multicomponent lifestyle intervention, a subsequent semen analysis in May 2026 detected spermatozoa within the severe oligozoospermic range, with measurable progressive motility and morphology. Total testosterone was within the reporting laboratory’s reference interval at the available follow-up assessment, while microbiological cultures showed no significant bacterial growth. These observations are descriptive and hypothesis-generating; the temporal sequence does not establish causality.

Participants were counselled to optimise sleep and stress management, progressively increase physical activity, avoid tobacco smoke, and minimise unnecessary pesticide or solvent exposure. Environmental exposures were not quantified, and adherence to this guidance was self-reported.

The lifestyle-medicine physician maintained daily contact with participants during the intensive phase to review their reported consumption of prescribed foods and adherence to the other lifestyle components. Adherence was therefore monitored closely but remained based on participant self-report and available contemporaneous clinical documentation. No validated adherence scale, objective dietary biomarker, weighed food record, repeated 24-hour dietary recall, or validated food-frequency questionnaire was used.

Complete participant-level quantitative dietary records were unavailable; consequently, retrospective 24-hour intake could not be reconstructed with sufficient reliability for quantitative analysis. To characterise the fixed minimum intensive-phase prescription, energy, protein, carbohydrate, total fat, saturated fat, fibre, and α-linolenic acid were estimated for the prescribed 454 g vegetable minimum combined with each permitted omega-3-source scenario (ground flaxseed, chia seeds, or flaxseed oil). McCance and Widdowson’s Composition of Foods Integrated Dataset (CoFID) 2021 (21) was used for the vegetable components. Because CoFID did not contain corresponding entries for ground flaxseed, chia seeds, or cold-pressed flaxseed oil, USDA FoodData Central was used for these components and for the household-measure-to-weight conversions (22). The resulting nutrient estimates are presented in **Supplementary Table 1**. These estimates describe the minimum prescribed protocol rather than participants’ total or actual dietary intake; ad libitum vegetables, fruit, and other variable foods were not included.

After spermatozoa were detected, participants transitioned to a maintenance phase involving gradual reintroduction of selected cooked whole-plant foods, additional fruit, legumes, whole grains, nuts and seeds while prescribed hydration and a substantial raw whole-plant component were retained.

### Diagnostic assessment

Before the intervention, each participant had at least one available semen-analysis report documenting that no spermatozoa were detected. Clinical records and participant histories also referred to additional results reported as azoospermia, although the number of original laboratory reports and the methodological detail available for retrospective review differed among the cases. Centrifugation and microscopic examination of the resulting sediment or pellet were recorded only when explicitly stated in the source document and were not inferred when these details were absent. In this case series, “reported azoospermia” therefore denotes a recorded laboratory finding of no spermatozoa; it does not indicate that WHO-concordant confirmation could be independently verified for every pre-intervention specimen (23).

Available clinical histories, semen-analysis reports, and fertility records were reviewed for evidence of obstruction. No obstructive lesion was documented; however, obstructive azoospermia could not be excluded because examination of the testes, epididymides and vasa deferentia, assessment of testicular volume, and clinically indicated reproductive-tract imaging were not documented consistently. The cases were therefore considered azoospermia of incompletely characterised type rather than classified definitively as obstructive or non-obstructive. Separately, karyotyping and Y-chromosome microdeletion testing were not performed or were not documented; consequently, genetic causes of impaired spermatogenesis could not be evaluated.

The available endocrine assessments comprised total testosterone, follicle-stimulating hormone (FSH), luteinising hormone (LH), and prolactin, although the panels differed between participants. Follow-up testing was available only for Case 1 (FSH, LH, total testosterone, and prolactin at approximately 10 weeks) and Case 3 (total testosterone and prolactin at approximately 10 weeks), precluding systematic comparison across cases. External laboratories were used, with results interpreted according to the reference intervals stated in the corresponding reports; results from different laboratories were not compared directly.

### Previous fertility-directed treatment and timeline reconstruction

Previous fertility-directed treatments and their timing were reconstructed from available clinical records and participant histories. Where exact medication-cessation dates were unavailable, the final documented or estimated fertility-treatment event was used; for Case 2, this was the August 2023 TESE. Treatment-to-program and treatment-to-detection intervals are presented in **Table 4** and are descriptive rather than formal pharmacological washout periods.

**Table 4:** Timeline from the final documented fertility-directed treatment or procedure to lifestyle-program initiation and subsequent detection of spermatozoa.

Case	Final documented fertility-directed treatment or procedure*	Lifestyle-program initiation	Approximate treatment-to-program interval‡	First semen analysis detecting spermatozoa	Approximate treatment-to-detection interval§
Case 1	Estimated cessation of fertility-directed pharmacotherapy, May 2024	22 April 2025	Approximately 11 months	1 July 2025	Approximately 14 months
Case 2	Testicular sperm extraction, August 2023	11 January 2026	Approximately 29 months	22 March 2026	Approximately 31 months
Case 3	Estimated cessation of fertility-directed pharmacotherapy, June 2023	16 March 2026	Approximately 33 months	25 May 2026	Approximately 35 months

*For Cases 1 and 3, the dates shown represent estimated cessation of fertility-directed pharmacotherapy based on available clinical records and participant histories; exact dates of final medication administration were unavailable. For Case 2, August 2023 represents the documented date of testicular sperm extraction (TESE), which marked the end of the documented fertility-treatment period, rather than a verified date of final hormonal medication administration.

**‡** The treatment-to-program interval denotes the period between the final documented or estimated fertility-directed treatment event and initiation of the multicomponent lifestyle programme. It should not be interpreted as a formal pharmacological washout period or as evidence that all biological effects of previous hormonal treatment had resolved.

**§** The treatment-to-detection interval denotes the period between the final documented or estimated fertility-directed treatment event and the first available semen analysis in which spermatozoa were detected. Because semen analyses were not performed continuously during the intervening periods, this interval does not establish the precise time at which spermatozoa first reappeared.

### Outcome assessment

The primary outcome was subsequent detection of spermatozoa after pre-intervention semen analyses had been reported as azoospermic. Secondary observations comprised available semen, endocrine and microbiological findings, limited weight and BMI measurements, and patient-reported changes summarised separately in Patient Perspectives. Body weight was extracted from clinical records without a prespecified serial measurement schedule.

## Case descriptions

### Case 1

Case 1 was a 38-year-old married man who presented with primary infertility of approximately three years’ duration. At least two previous semen analyses had reported azoospermia, including analyses of centrifuged specimens in which no spermatozoa were identified. He had no history of conception, testicular trauma, cryptorchidism, genitourinary infection, scrotal surgery, exogenous testosterone use, chemotherapy exposure, or a known genetic disorder. At the available assessment on 25 April 2024, his weight was 100.6 kg and his BMI was 34.8 kg/m², within the obesity range. Other relevant clinical features included central adiposity, hypertension, obstructive sleep apnoea, chronic fatigue, and reduced exercise tolerance. He also reported frequent consumption of ultra-processed foods and alcohol, together with a low intake of fruits and vegetables.

He reported high programme adherence, although this was not objectively verified, together with improved sleep practices, increased physical activity and substantially reduced alcohol consumption.

Fertility-directed pharmacotherapy was estimated to have ceased in May 2024. The lifestyle programme began on 22 April 2025, giving an estimated treatment-free interval of approximately 11 months. No concurrent fertility-directed hormonal therapy was reported during the intervention. Spermatozoa were detected in the ejaculate on 1 July 2025, approximately 10 weeks after programme initiation and 14 months after estimated medication cessation.

Spermatozoa were detected on 1 July 2025 at 1.6 × 10^6^/mL and remained detectable at 1.4 × 10^6^/mL on 3 March 2026, within the severe oligozoospermic range at both assessments. After the intensive phase, the participant reported resuming episodic binge alcohol consumption; its relationship to subsequent semen variation could not be determined. Complete longitudinal findings are presented in **Table 1**.

Between 25 April 2024 and the first sperm-positive assessment on 1 July 2025, his weight decreased from 100.6 to 88.0 kg, with BMI falling from 34.8 to 30.5 kg/m². Following the first sperm-positive analysis, the participant entered the maintenance phase. Maintenance-phase adherence was based on self-report and was not objectively verified. Serial anthropometric and metabolic measurements were not obtained consistently, and no major adverse events were documented during follow-up.

### Case 2

Case 2 was a 40-year-old married man who presented with primary infertility of approximately five years’ duration. Available fertility records reported azoospermia on multiple semen analyses. Post-centrifugation assessment was documented only for those analyses whose source laboratory reports explicitly recorded this procedure. At the available assessment on 23 June 2023, his weight was 89.7 kg and his BMI was 29.3 kg/m², within the overweight range. Relevant medical and lifestyle history included reduced physical activity, poor sleep quality, low energy levels, and longstanding consumption of ultra-processed foods.

Before entering the programme, the participant had received clomiphene citrate, human chorionic gonadotropin, human menopausal gonadotropin, antioxidants and other fertility-directed therapies; exact dosing and treatment schedules were unavailable. Testicular sperm extraction in August 2023 yielded no viable spermatozoa for assisted reproduction and was used as the final documented fertility-treatment event. Available semen analyses remained reported as azoospermic before programme initiation.

The lifestyle programme began in mid-January 2026, corresponding to an estimated treatment-to-intervention interval of approximately 29 months. Spermatozoa were first detected in the ejaculate on 22 March 2026, approximately 10 weeks after programme initiation and 31 months after the August 2023 TESE, which represented the final documented fertility-directed treatment event. No concurrent fertility-directed hormonal treatment was reported during the lifestyle programme.

Semen analysis on 22 March 2026 detected spermatozoa at 2.0 × 10^6^/mL, within the severe oligozoospermic range; complete semen parameters are presented in **Table 2**.

Seminal culture on 1 April 2026 showed heavy growth of *Staphylococcus aureus*; its relationship to the semen findings is uncertain (**Table 2**).

Between 23 June 2023 and the first sperm-positive assessment on 22 March 2026, his weight decreased from 89.7 to 81.7 kg, corresponding to a reduction in BMI from 29.3 to 26.7 kg/m². Following the first sperm-positive analysis, the participant entered the maintenance phase. No major adverse events were documented during follow-up.

### Case 3

Case 3 was a 36-year-old married man who presented with primary infertility of approximately three years’ duration. Semen analysis on 17 February 2023 showed severe oligozoospermia, with a sperm concentration of 0.1 × 10^6^/mL. A subsequent analysis performed on 6 March 2023 reported no spermatozoa detected. He had no history of conception, cryptorchidism, testicular trauma, genitourinary surgery, exogenous testosterone use, or chemotherapy exposure. At the available assessment on 6 March 2023, his weight was 86.8 kg and his BMI was 29.0 kg/m², within the overweight range. His medical and lifestyle history was also notable for sedentary behaviour, irregular sleep, low physical activity, intermittent fatigue, frequent consumption of processed foods, and limited intake of minimally processed plant foods.

Before entering the lifestyle programme, the participant reported receiving fertility-directed hormonal therapy, antioxidant supplements, and herbal preparations. Subsequent semen testing continued to report azoospermia. Fertility-directed pharmacotherapy was estimated to have ceased in June 2023. Although he had been considering surgical sperm retrieval, he elected to undertake the lifestyle programme before proceeding with surgery. The programme began on 16 March 2026, corresponding to an estimated treatment-to-program interval of approximately 33 months. No concurrent fertility-directed hormonal therapy was reported during the programme.

The participant reported generally good adherence during follow-up, although adherence was based on self-report. Semen analysis on 25 May 2026, approximately 10 weeks after programme initiation and 35 months after estimated cessation of fertility-directed pharmacotherapy, detected spermatozoa at 2.2 × 10^6^/mL, within the severe oligozoospermic range. Complete semen, endocrine and microbiological findings are presented in **Table 3**. Between 6 March 2023 and the first sperm-positive assessment on 25 May 2026, his weight decreased from 86.8 to 77.8 kg, corresponding to a reduction in BMI from 29.0 to 26.0 kg/m². No major adverse events were documented. Because no subsequent semen analysis was available for verification against an original source report, persistence of detectable spermatozoa beyond 25 May 2026 could not be established.

### Patient perspectives

Participants reported improvements in energy, sleep quality, physical-activity tolerance, libido, and general well-being during follow-up. These outcomes were self-reported, were not assessed using validated instruments, and cannot be attributed independently to the intervention. No participant reported a major adverse event during the documented follow-up period.

## Discussion

The principal observation in this case series was the detection of spermatozoa during follow-up in three men whose available pre-intervention records reported azoospermia; concentrations remained within the severe oligozoospermic range. Because the series was uncontrolled and aetiological assessment, semen-analysis methods, previous-treatment timing and intervention adherence were incompletely standardised, causality cannot be inferred. The recurrent temporal pattern is therefore hypothesis-generating and warrants prospective controlled investigation.

Because the intervention was multicomponent, weight loss is a plausible co-intervention rather than a background variable. Obesity and related metabolic dysfunction may adversely affect spermatogenesis through endocrine dysregulation, systemic inflammation, oxidative stress, and testicular metabolic or lipotoxic pathways (24–26). Diet-induced weight loss has been associated with increased sperm concentration and total sperm count in men with obesity, but this does not demonstrate restoration of spermatogenesis in azoospermia (27). Increased physical activity and reduced high alcohol exposure are also recognised as modifiable factors relevant to male fertility (13, 14). Changes in sleep and stress management may have contributed but were not objectively quantified.

Total daily energy and nutrient intake was not measured systematically. **Supplementary Table 1** presents modelled estimates of the energy and nutrient composition of the fixed minimum food prescription; it excludes ad libitum vegetables, fruit, other permitted foods, and supplements. It therefore cannot be used to estimate participants’ total energy, macronutrient, or micronutrient intake or the magnitude of dietary energy restriction during the intervention.

The reductions between the available body-weight measurements are compatible with a modest average negative energy balance across the corresponding intervals. Using the conventional approximation of 7,700 kcal per kilogram of weight loss, the estimated mean energy deficits across the intervals between available weight measurements were approximately 225 kcal/day over 62 weeks for Case 1, 61 kcal/day over 143 weeks for Case 2, and 59 kcal/day over 168 weeks for Case 3 (28). These estimates are retrospective approximations rather than direct measurements of dietary intake or intervention-related caloric restriction. Because weight was not recorded serially and most of each measurement interval preceded programme initiation, the timing and trajectory of weight loss are unknown; periods of greater negative energy balance may have alternated with weight stability or partial regain. Moreover, the energy content of weight loss varies over time and with changes in fat and fat-free mass, so the 7,700-kcal/kg approximation should be interpreted cautiously (28). Dietary modification occurred alongside weight loss and other behavioural changes, and mechanistic biomarkers were not assessed systematically; consequently, the contribution of individual programme components cannot be determined.

### Comparison with the mediterranean dietary pattern

Because much of the clinical dietary literature in male fertility concerns Mediterranean dietary patterns, the intensive-phase protocol warrants comparison with—but should not be conflated with—a conventional Mediterranean diet. It consisted primarily of raw leafy green and cruciferous vegetables, limited fruit intake, and prescribed sources of α-linolenic acid, while excluding animal-derived foods, legumes, nuts, grains, refined foods, and ultra-processed foods. Studies of Mediterranean dietary patterns have reported generally favourable, although inconsistent, associations with semen parameters, but there is no evidence that these diets restore spermatogenesis in men with azoospermia (29–32). Mediterranean-diet studies therefore provide only indirect context and should not be interpreted as evidence for the effectiveness of the specific intervention used in this case series.

### Previous treatment and variability in semen findings

Beyond uncertainty regarding the contribution of individual programme components, previous fertility-directed treatment remains an important alternative explanation. Treatment-to-program intervals were approximately 11, 29 and 33 months for Cases 1–3, respectively, with spermatozoa first detected approximately 14, 31 and 35 months after the corresponding reference events (**Table 4**). These intervals make a direct short-term carryover effect less plausible, but delayed or interacting treatment effects and earlier unobserved reappearance of spermatozoa cannot be excluded because exact cessation dates and continuous semen surveillance were unavailable.

Semen trajectories were neither uniform nor linear and may have been influenced by biological variation, ejaculatory abstinence interval, behavioural exposures, and inter-laboratory or operator variability (33–35). In Case 1, resumption of episodic alcohol consumption coincided with variation in semen findings, but this temporal association does not establish causality. These uncertainties reinforce the need for repeated semen analyses under standardised conditions with documentation of abstinence interval, sample collection, concurrent exposures, previous treatment and intervention adherence.

Several limitations constrain interpretation. Most importantly, body weight, hormonal parameters and dietary intake were not recorded systematically before and during the intervention in all three participants. Only isolated weight measurements and non-uniform endocrine panels were available, while dietary exposure and adherence were self-reported. No repeated 24-hour dietary recalls, weighed records, validated food-frequency questionnaire or objective dietary biomarkers were used. **Supplementary Table 1** therefore characterises only the fixed minimum prescription and cannot quantify actual energy or nutrient intake, reconstruct weight-loss dynamics or support comparisons across intervention phases. Consequently, the dietary, metabolic, hormonal or lifestyle factors contributing to subsequent sperm detection cannot be distinguished.

The uncontrolled design also precludes causal inference and is vulnerable to biological, sampling and laboratory variation, regression to the mean, residual confounding and delayed effects of previous fertility treatment. WHO-concordant confirmation of every pre-intervention azoospermic specimen was unavailable. Obstruction could not be excluded because relevant examination and imaging were inconsistently documented, while karyotyping and Y-chromosome microdeletion testing were unavailable. Mechanistic biomarkers and reproductive outcomes, including conception and live birth, were not assessed systematically. These cases therefore document a temporal transition to detectable spermatozoa, not normalisation of fertility or treatment efficacy.

## Conclusion

In this three-case series, spermatozoa were detected during follow-up in all three men whose available pre-intervention records had reported azoospermia, approximately 10 weeks after initiation of a structured multicomponent lifestyle programme. Although sperm concentrations remained within the severe oligozoospermic range, the consistent temporal transition from no spermatozoa reported before intervention to detectable spermatozoa is clinically noteworthy. These observations cannot establish causality or determine the contribution of diet, weight loss, physical activity, sleep, alcohol reduction, or other programme components. They nevertheless identify a clinically relevant and understudied question: whether modification of metabolic and lifestyle factors can influence spermatogenic function in selected men with azoospermia. Prospective controlled studies incorporating WHO-standardised semen assessment, comprehensive andrologic and aetiological evaluation, objective adherence measures, and clinically meaningful reproductive outcomes are warranted.

## Data Availability

The original contributions presented in the study are included in the article/**Supplementary Material**. Further inquiries can be directed to the corresponding author.
