# Supplementary material for: Case Report: Reappearance of spermatozoa following multicomponent lifestyle modification in men with previously diagnosed azoospermia: a case series
Source: Front Urol. 2026 Sep 28;6:1903697. doi: 10.3389/fruro.2026.1903697 (PMC13662741; doi:10.3389/fruro.2026.1903697)
Supplement: Supplementary file 1 [file Table1.docx]

**Supplementary Table S1. Estimated nutrient composition of the fixed minimum daily food prescription during the intensive phase under three permitted omega-3-source scenarios**

**Interpretive scope.** These values model the prescribed minimum protocol; they are not estimates of any participant’s total or actual intake. The 454 g vegetable minimum is represented by an equal-weight six-vegetable mixture. Ad-libitum vegetables, fruit, other permitted foods and supplements are excluded because quantities were not recorded consistently.

**Panel A. Modelled daily food quantities**

| **Protocol component** | **Ground-flaxseed scenario** | **Chia-seed scenario** | **Flaxseed-oil scenario** |
| --- | --- | --- | --- |
| Raw mature spinach (CoFID 13-572) | 75.7 g | 75.7 g | 75.7 g |
| Raw curly kale (CoFID 13-234) | 75.7 g | 75.7 g | 75.7 g |
| Raw average cabbage (CoFID 13-582) | 75.7 g | 75.7 g | 75.7 g |
| Raw rocket/arugula (CoFID 13-522) | 75.7 g | 75.7 g | 75.7 g |
| Raw cauliflower (CoFID 13-512) | 75.7 g | 75.7 g | 75.7 g |
| Raw green broccoli (CoFID 13-502) | 75.7 g | 75.7 g | 75.7 g |
| Total leafy-green/cruciferous vegetable mixture | 454 g | 454 g | 454 g |
| Ground flaxseed | ½ cup (56 g) | — | — |
| Chia seeds | — | ½ cup (84 g) | — |
| Cold-pressed flaxseed oil | — | — | 3 tbsp (40.8 g) |
| Water | 3.0–3.8 L | 3.0–3.8 L | 3.0–3.8 L |

*Model assumption: calculations use exactly 454/6 = 75.6667 g of each vegetable; 75.7 g is shown after rounding. The equal-weight blend is a transparent analytical convention and does not imply that every participant consumed each vegetable daily.*

**Panel B. Estimated energy and macronutrient composition**

| **Protocol component** | **Ground-flaxseed scenario** | **Chia-seed scenario** | **Flaxseed-oil scenario** |
| --- | --- | --- | --- |
| **Energy (kcal)** | 424.6 | 533.8 | 486.3 |
| **Protein (g)** | 24.2 | 27.8 | 14.0 |
| **Available carbohydrate (g)** | 12.2 | 17.8 | 11.4 |
| **Fibre (g; mixed definitions)¹** | 25.2 | 38.8 | 9.9 |
| **Total fat (g)** | 26.6 | 28.8 | 43.8 |
| **Saturated fatty acids (g)** | 2.5 | 3.3 | 4.1 |
| **Polyunsaturated fatty acids (g)** | 17.7 | 21.5 | 29.3 |
| **Alpha-linolenic acid, ALA (g)²** | 12.8 | 15.0 | 21.8 |

**Panel C. Estimated mineral composition**

| **Protocol component** | **Ground-flaxseed scenario** | **Chia-seed scenario** | **Flaxseed-oil scenario** |
| --- | --- | --- | --- |
| **Sodium (mg)** | 195 | 192 | 179 |
| **Potassium (mg)** | 2107 | 1994 | 1652 |
| **Calcium (mg)** | 625 | 1012 | 482 |
| **Magnesium (mg)** | 343 | 405 | 123 |
| **Phosphorus (mg)** | 587 | 949 | 227 |
| **Iron (mg)** | 8.5 | 11.8 | 5.3 |
| **Zinc (mg)** | 4.5 | 5.9 | 2.1 |
| **Selenium (µg)** | 20.3 | 52.4 | 6.1 |

**Panel D. Estimated selected vitamin composition**

| **Protocol component** | **Ground-flaxseed scenario** | **Chia-seed scenario** | **Flaxseed-oil scenario** |
| --- | --- | --- | --- |
| **Vitamin C (mg)** | 257 | 258 | 257 |
| **Folate (µg)** | 455 | 447 | 406 |
| **Vitamin E, alpha-tocopherol (mg)** | 4.3 | 4.6 | 4.4 |
| **Vitamin K1 (µg; minimum estimate)³** | ≥1050 | ≥1047 | ≥1051 |

**Calculation notes**

| **1.** | CoFID AOAC fibre values were unavailable for raw mature spinach and raw curly kale. Vegetable fibre was therefore calculated using CoFID non-starch polysaccharide (NSP) values, while flaxseed and chia used USDA total dietary fibre. The combined fibre values are approximate and should not be interpreted as methodologically uniform. |
| --- | --- |
| **2.** | ALA values represent the prescribed flaxseed, chia or flaxseed-oil component only. CoFID did not provide directly comparable ALA values for every selected vegetable; vegetable ALA was therefore not added. |
| **3.** | CoFID did not report vitamin K1 for raw rocket. The displayed vitamin K1 values therefore exclude rocket and are minimum estimates. The chia value also excludes chia vitamin K1 because it was unavailable in the USDA entry used for nutrient calculations. |
| **4.** | CoFID ‘trace’ values were treated as zero in arithmetic. Available carbohydrate for vegetables used the CoFID carbohydrate field; for flaxseed and chia it was calculated as USDA carbohydrate by difference minus total dietary fibre. |
| **5.** | Fruit was limited to approximately 25% of daily food volume but was not quantified. Ad-libitum vegetables and other permitted foods were also not recorded systematically; none are included in these estimates. |
| **6.** | Household conversions: ground flaxseed, 1 tbsp = 7 g (8 tbsp = ½ cup = 56 g); chia, 1 cup = 168 g (½ cup = 84 g); flaxseed oil, 1 tbsp = 13.6 g (3 tbsp = 40.8 g). |

**Data sources**

• UK Department of Health and Social Care. Composition of Foods Integrated Dataset (CoFID), 2021. Selected raw vegetable codes: 13-572, 13-234, 13-582, 13-522, 13-512 and 13-502. https://www.gov.uk/government/publications/composition-of-foods-integrated-dataset-cofid

• US Department of Agriculture, FoodData Central. Seeds, flaxseed (FDC 169414); seeds, chia seeds, dried (FDC 170554); oil, flaxseed, cold pressed (FDC 167702); chia portion conversion (FDC 2707590). https://fdc.nal.usda.gov/

*Abbreviations: ALA, alpha-linolenic acid; CoFID, Composition of Foods Integrated Dataset; NSP, non-starch polysaccharides; USDA, United States Department of Agriculture.*
